# Ab initio investigation of functionalization of titanium carbide Ti_3_C_2_ MXenes to tune the selective detection of lung cancer biomarkers

**DOI:** 10.1038/s41598-024-51692-6

**Published:** 2024-01-16

**Authors:** Wadha Alfalasi, Tanveer Hussain, Nacir Tit

**Affiliations:** 1 Department of Physics, College of Science, UAE University, P.O. Box 15551, Al-Ain, United Arab Emirates; 2 National Water and Energy Center, UAE University, P.O. Box 15551, Al-Ain, United Arab Emirates; 3 School of Science and Technology, University of New England, Armidale, NSW 2351 Australia

**Keywords:** Biophysics, Cancer, Computational biology and bioinformatics, Biomarkers, Diseases, Health care, Medical research, Signs and symptoms

## Abstract

Selected volatile organic compounds (VOCs), such as benzene (C_6_H_6_), cyclohexane (C_6_H_12_), isoprene (C_5_H_8_), cyclopropanone (C_3_H_4_O), propanol (C_3_H_8_O), and butyraldehyde butanal (C_4_H_8_O), in exhaled human breath can act as indicators or biomarkers of lung cancer diseases. Detection of such VOCs with low density would pave the way for an early diagnosis of the disease and thus early treatment and cure. In the present investigation, the density-functional theory (DFT) is applied to study the detection of the mentioned VOCs on Ti_3_C_2_T_X_ MXenes, saturated with the functional groups T_x_ = O, F, S, and OH. For selectivity, comparative sensing of other interfering air molecules from exhaled breath, such as O_2_, N_2_, CO_2_, and H_2_O is further undertaken. Three functionalization (T_x_ = O, F, and S) are found promising for the selective detection of the studied VOCs, in particular Ti_3_C_2_O_2_ MXenes has shown distinct sensor response toward the C_5_H_8_, C_6_H_6_, C_6_H_12_, and C_3_H_4_O. The relatively strong physisorption (${E}_{ads}\cong -0.45 to-0.65 {\text{eV}}$), triggered between VOC and MXene due to an enhancement of van der Waals interaction, is found responsible to affect the near Fermi level states, which in turn controls the conductivity and consequently the sensor response. Meanwhile, such intermediate-strength interactions remain moderate to yield small desorption recovery time (of order $\tau \cong \mu {\text{s}}-{\text{ms}})$ using visible light at room temperature. Thus, Ti_3_C_2_O_2_ MXenes are found promising candidate material for reusable biosensor for the early diagnosis of lung cancer diseases through the VOC detection in exhaled breath.

## Introduction

According to the World Health Organization, lung cancer (LC) accounted for 2.21 million new cases of cancer globally in 2020, making it the second most common cancer (after breast cancer) and the leading cause of cancer deaths^1^. Compared to LC diagnosed at a more advanced stage, early diagnosis improves survival and is crucially important for the start of a successful medical therapy and plausible high chance of curing before its spread in human body^2^. Different investigations of early-stage cancer have shed light on the alterations that take place during the early phases of tumor formation^2,3^. It was proven that for many cancer diseases, such as liver, lung, bowl and breast cancers, physiotherapeutic treatments exist provided early prognoses are achieved on time^4–6^. For instance, early diagnosis may yield good chance of survival ranging from 6/10 in lung cancer, to 9/10 in bowel cancer, and to 10/10 in breast cancer^6^.

More than half of number of patients with LC die within a year after being diagnosed, making it one of the leading causes of cancer deaths^7^, as attributed to the challenges in the therapy and diagnosis. The additional complications and high costs of examinations involving bronchoscopy and needle biopsies make them unsuitable for population screening^8^. Towards reducing the patient’s mortality rate, early diagnosis of cancer through applicable and non-invasive techniques is vital, such as blood based liquid biopsies in capturing the circulating biomarkers^9^. Another non-invasive approach in the detection of cancers in the early stage is through the analysis of volatile organic compounds (VOCs) biomarkers existing within the exhaled breath of the patients^9,10^ which should be more reliable as it is easy and cost effective. Statistics shows that only 16% of lung cancers can be detected at early stage^7^. From perspectives of VOCs’ analysis, in 1971, Linus Pauling discovered that healthy human breath contains about 200 VOCs^11^. Hence, the question remains to which appropriate VOCs to select as lung-cancer biomarkers and beyond what critical density to decide about the cancer diagnosis, and finally what materials should be suitable for the detection.

In a statistical study, Michalis Koureas and co-workers^3^ investigated the prospect of using breath analysis to differentiate between LC, other lung diseases, and the healthy control group. The levels of 19 VOCs were measured in research participants' exhaled breath. Significant differences in several substances between LC patients and healthy controls were discovered. Exogenous monoaromatic, 1- and 2-propanol biomarker sets predominately and effectively distinguished between LC patients and healthy controls. The quantities of these substances in the patient's breath may indicate changes in their physiological and biochemical status, and they may be employed as probes to examine LC^3^.

In another related work, Reji and coworkers^12^ used density functional theory (DFT) calculations and presented a comparative adsorption study of six VOCs biomarkers (i.e., acetone, ethanol, acetonitrile, 2-propanol, isoprene, and toluene) on 2D Sc_2_CO_2_ MXenes nanosheet. The authors reported that all VOCs can be detected via chemiresistive mechanism; while the weakly adsorbed species (e.g., toluene and isoprene) can also be detected based on the change in the work functions. Moreover, Wan and coworkers^13^ employed first-principles DFT calculations for studying the comparative adsorption of six VOCs biomarkers (i.e., isoprene “C_5_H_8_”, methyl cyclopentane “C_6_H_12_”, 1-propanol “C_3_H_8_O”, 2-propenal “C_3_H_4_O”, benzene “C_6_H_6_”, and styrene “C_8_H_8_”) on TM-doped transition-metal di-chalcogenides (TMDs) monolayer (namely, Ru-doped SnS_2_). Selective chemisorptions of three VOCs were reported (namely, C_3_H_4_O, C_5_H_8_O, and C_6_H_6_), and consequently corroborating the suitability and the selectivity of the studied TMDs towards VOCs lung cancer biomarkers.

Exploring defects versus doping in blue phosphorene (BlueP) to simulate experimental data, Sun and coworkers^14^ used DFT to study the interaction mechanism towards the capture of some selected VOCs, such as acetone, ethanol and propanal. They focused on defects like single and multiple vacancies and on dopants like S/Si. They reported that only mono-vacancy and S-substitutional doping to have great potential for the detection of their studied VOCs. In a further exploration of 2D materials for the detection of biomarkers, Hussain and coworkers^15^ presented a spin-polarized DFT study on nitogenated holey graphene (C_2_N), graphdiyne (GDY), and their hetero-structure (C_2_N∙∙∙GDY) to detect selected VOCs, such as acetone, ethanol, propanal, and toluene. They found that the incorporation of C_2_N in hetero-structure (C_2_N∙∙∙GDY) is necessary to enhance the van der Waals interactions with VOCs. They further applied thermodynamic analysis to study the sensing characteristics of VOCs under ambient conditions. They proposed C_2_N∙∙∙GDY hetero-structure as promising material for sensing certain VOCs.

After the breakthrough synthesis of MXenes by Yuri Gogotsi and coworkers in 2011^16,17^, these 2D materials subsequently found their applications in several fields, such as gas-sensing^18–21^, biosensing^12,22^, energy-storage^23^, and metal-ion batteries^24,25^. Since their invention date, Ti_3_C_2_T_x_ MXenes^16,17^ have been well characterized both experimentally^26^ and theoretically^27^ by being thermodynamically very stable. For instance, recently, Lu et al.^27^ presented a combination of experimental and simulation studies of Ti_3_C_2_T_x_ MXenes (T = F, OH, and O) to demonstrate their relevance for energy storage applications. These authors^27^ showed the phonon spectra lacking negative frequencies as an evidence of structural stability of these MXenes. Furthermore, the distinguished and unique characteristics of MXenes’ families stem in the moderate interaction (predominantly van der Waals type) between the incident molecules and the polar surface of the functionalized MXenes. Such moderate interaction scales between strong physisorption, with ability to induce electric dipole moments in small molecules, such as in hydrogen molecules and thus making MXenes suitable for hydrogen storage, to weak chemisorption with metallic compounds making MXenes suitable for battery applications^24,28^. Hence, in the context of biomarker detection, it’s worth noting that VOCs consist of organic molecules that are slightly larger than the small air interfering molecules of exhaled human breath. Consequently, MXenes have the potential to induce dipole moments in various regions of VOCs, leading to stronger physisorption compared to other ambient air molecules. Such characteristics renders MXenes well-suited for selective sensing of VOCs^12^. Nevertheless, the ongoing challenge is to evaluate and optimize the ideal passivation layer that can further enhance the selectivity for VOCs specific for LC diseases.

The scope of the present investigation is to employ the state-of-the-art DFT technique by using the Vienna Ab-initio Simulation Package (VASP) to study five Ti_3_C_2_T_x_ {T_x_ = O, F, S, (OH), and F(OH)} MXenes for the selective detection of six representative VOCs as lung-cancer biomarkers, such as C_5_H_8_, C_6_H_6_, C_6_H_12_, C_3_H_4_O, C_3_H_8_O, and C_4_H_8_O. Contrasted sensing mechanism of four interfering air molecules, such as O_2_, N_2_, CO_2_, and H_2_O from the exhaled healthy human breath have also been investigated. The study also comprises the electronic, magnetic, transport properties, and sensor response to explore the sensitivity and selectivity of the studied MXenes.

## Results and discussion

### Structural properties

**Figure 1:**
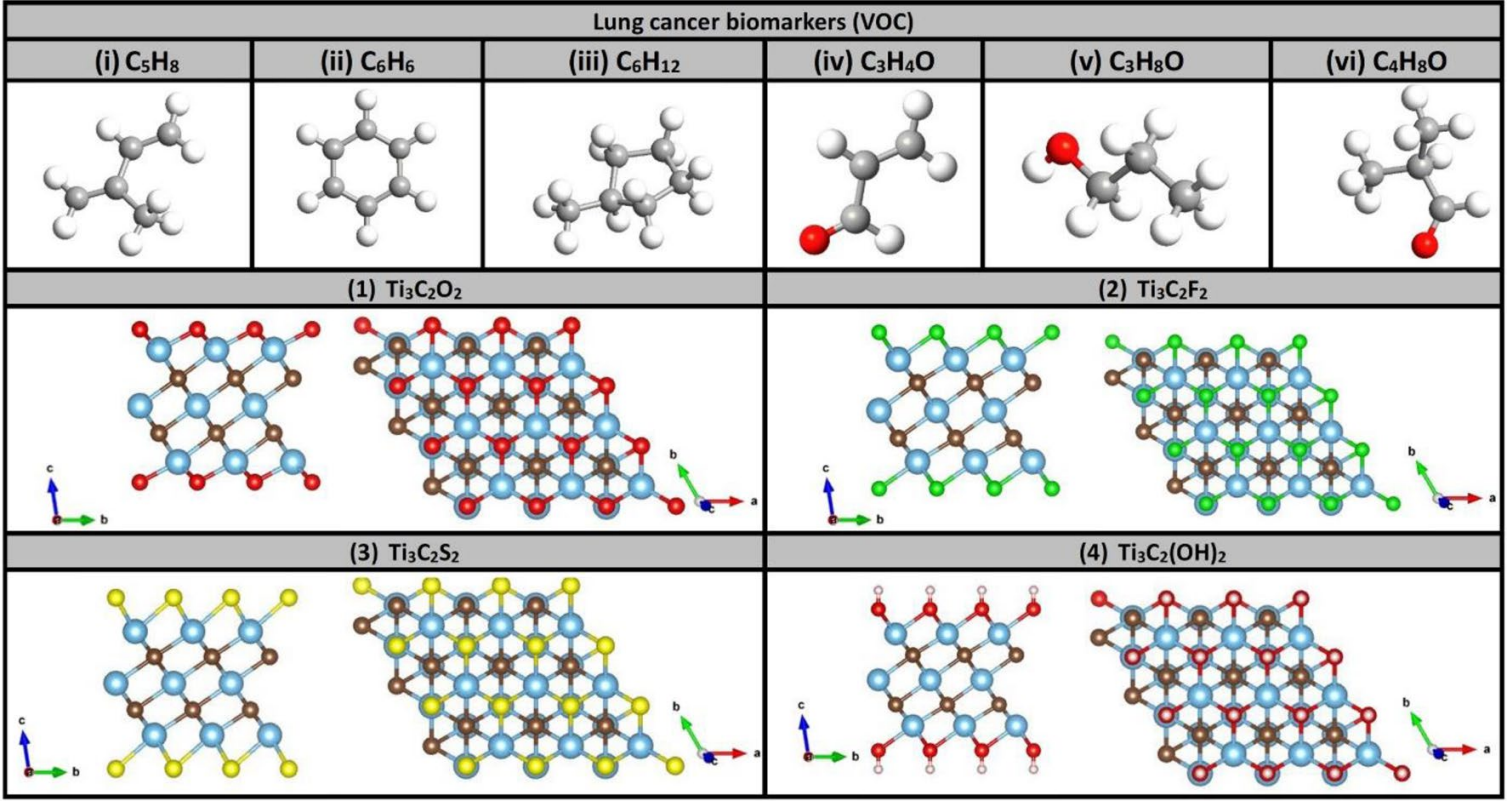
Relaxed atomic structures of six VOCs biomarkers in their free-standing states, and four Ti_3_C_2_T_x_ MXenes functionalized with T = O, F, S, and OH group, respectively. Atom Colors: C (grey), H (white), Ti (orange), O (red), F (green), and S (yellow). (**a**) Ti_3_C_2_O_2_, (**b**) Ti_3_C_2_F_2_, (**c**) Ti_3_C_2_S_2_, and (**d**) Ti_3_C_2_(OH)_2_. It should be emphasized that these structures have been experimentally synthesized and proven to be thermodynamically stable. As described in the previous section, the relaxed structures are in good agreement with literature as well^18^.

Figure 1 shows the relaxed structures before the adsorption processes. Top panels show the atomic structures of the 6 VOCs biomarkers, which were selected as lung cancer biomarkers. The relaxed structures of these VOCs are in good agreement with literature^13^. Lower four panels in Fig. 1 display the relaxed structures of pristine MXenes monolayers samples of: (1) Ti_3_C_2_O_2_, (2) Ti_3_C_2_F_2_, (3) Ti_3_C_2_S_2_, and (4) Ti_3_C_2_(OH)_2_ MXenes.

Furthermore, supported by the results of the electronic structure calculations below, these MXenes are paramagnetic having metallic characters. Their discrepancies are due to the passivation layers. Definitely, the passivation layers have great ability to make different populations of electric dipoles. The strongest dipoles should be attributed to the oxygen passivation. The electronegativity characters in descending order of strengths are: χ^F^ = 3.98 > χ^O^ = 3.44 > χ^S^ = 2.58 Pauling^29^. Although F atom is more electronegative than O atom, in passivation layer, oxygen is divalent and having a coordination of 2 whereas fluorine is monovalent and having same coordination of 2. Such coordination yielded transfer of charge to oxygen more than that to fluorine (i.e., |q(O)|= 1.065 >|q(F)|= 0.692 >|q(S)|= 0.352). Consequently, the electric dipole moments originating on oxygen atoms are stronger than those on fluorine. Thus, the vdW interactions of oxygen passivation should be justified to be the strongest.

### Adsorption properties

**Figure 2:**
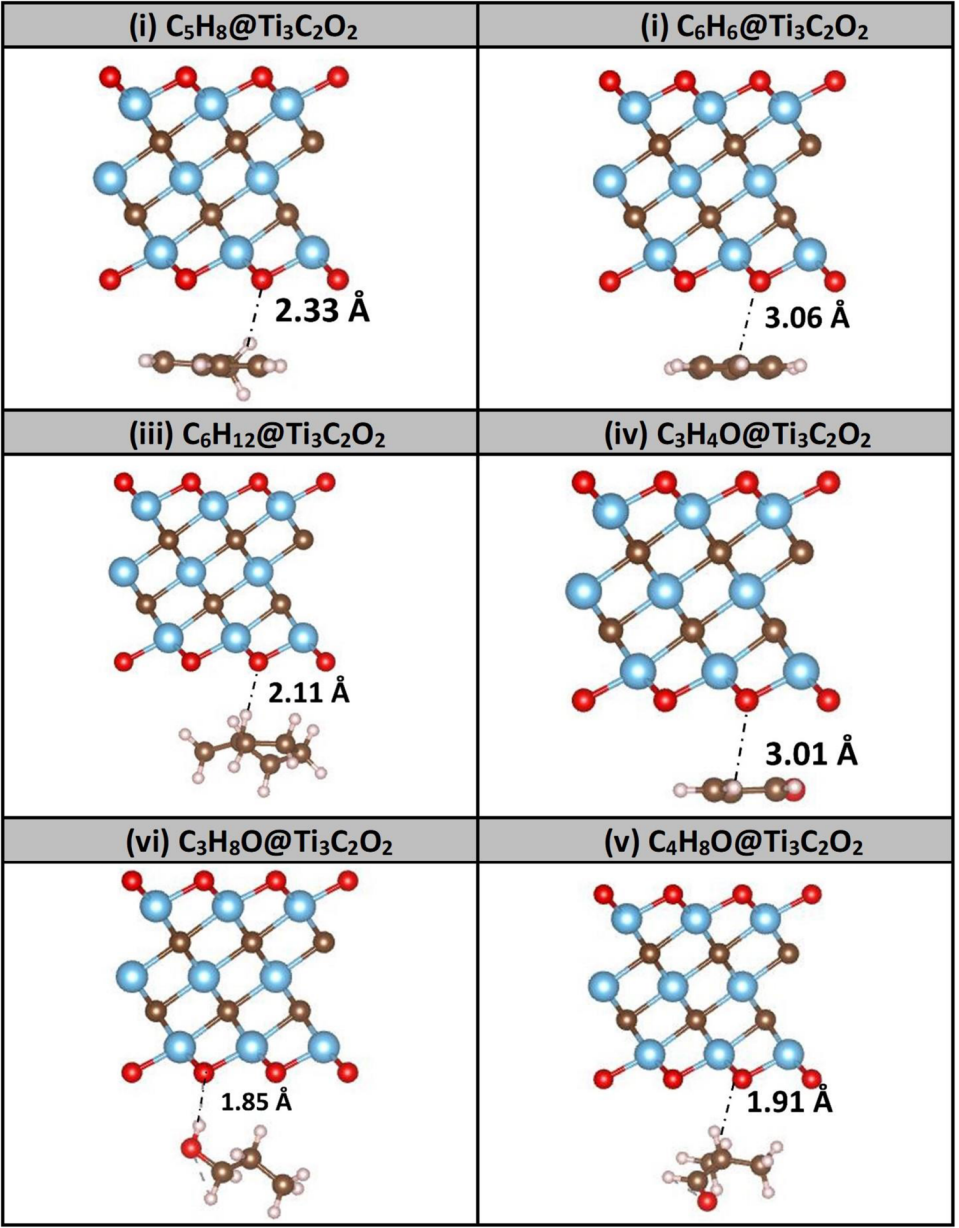
Relaxed lung cancer VOCs on the surface of Ti_3_C_2_O_2_ MXenes. Oxygen-based VOCs shows stronger physisorption as they possess stronger electric dipole moments. Colors: C (brown), Ti (blue), O (red), and H (purple).

For the sake of selective gas sensing, the adsorptions of the six VOCs lung-cancer biomarkers are studied and compared to the adsorptions of other four interfering air molecules existing in the exhaled human breath (i.e., N_2_, O_2_, CO_2_, and H_2_O). Actually, the adsorption of the whole 10 molecules on five different MXenes systems were assessed: (i) Ti_3_C_2_O_2_, (ii) Ti_3_C_2_F_2_, (iii) Ti_3_C_2_S_2_, (iv) Ti_3_C_2_(OH)_2_, and (v) Ti_3_C_2_F(OH). The results of adsorption energies, VOC-substrate distances, charge transfers, and magnetizations for six VOCs biomarkers are shown in Table 1. On the other hand, Table 2 displays the results of adsorption energies of the interfering air molecules and compare them to those ab-initio results existing in literature for sake of benchmarking. The results of Table 1 clearly corroborate the relevance of three functionalized MXenes towards the adsorption selectivity of biomarkers (namely, O–, F–, and S–passivated Ti_3_C_2_ MXenes). It should be emphasized that MXenes (iv-v) (i.e., Ti_3_C_2_(OH)_2_ and Ti_3_C_2_F(OH)) demonstrated complete lack of selectivity. Besides, one noticed peculiar behaviors of oxygen molecule as to chemically react on the surfaces of these latter MXenes. For instance, on the surface of Ti_3_C_2_F(OH) MXenes, O_2_ molecule attracts two hydrogen atoms and forms hydrogen peroxide H_2_O_2_. So, these configurations are excluded from further consideration. The relaxed structures due to the adsorption of 6 VOCs on particularly the Ti_3_C_2_O_2_ MXenes are displayed in Fig. 2. While all the VOCs exhibit physisorption processes, those containing oxygen exhibit the strongest interactions as displayed by the close distances to the MXenes’ surfaces; and they should consist best candidate biomarkers.

**Table 1:** Results of adsorption energy (E_ads_), VOCs-substrate relaxed distance (d), charge transfer (Δq), and magnetization (M) after relaxation of six VOC lung-cancer biomarkers on five Ti_3_C_2_T_x_ functionalized with T = O, F, S, (OH), or F(OH) groups.

	Ti_3_C_2_O_2_ (M_O_ = 0.00 μ_B_)				Ti_3_C_2_F_2_ (M_O_ = 0.00 μ_B_)				Ti_3_C_2_S_2_ (M_O_ = 0.00 μ_B_)				Ti_3_C_2_(OH)_2_ (M_O_ = 0.97 μ_B_)				Ti_3_C_2_F(OH)_2_ (M_O_ = 0.00 μ_B_)			
	E_ads_ (Ev)	d (Å)	∆q (e)	M(μ_B_)	E_ads_ (Ev)	d (Å)	∆q (e)	M(μ_B_)	E_ads_ (Ev)	d (Å)	∆q (e)	M(μ_B_)	E_ads_ (Ev)	d (Å)	∆q (e)	M(μ_B_)	E_ads_ (Ev)	d (Å)	∆q (e)	M(μ_B_)
C_5_H_8_	− 0.647	2.33	0.056	0.00	− 0.551	2.08	0.030	0.00	− 0.566	2.77	− 0.129	0.00	− 0.923	2.11	− 0.182	1.11	− 0.741	2.15	0.122	0.00
C_6_H_6_	− 0.586	3.0 +	0.051	0.00	− 0.483	3.04	0.059	0.00	− 0.574	3.28	− 0.0934	0.00	− 0.713	2.28	0.028	0.77	− 0.622	2.65	0.203	0.00
C_6_H_12_	− 0.582	2.11	− 0.14	0.00	− 0.499	2.39	− 0.071	0.00	− 0.576	2.32	− 1.365	0.00	− 0.549	1.20	− 0.089	0.48	− 0.577	1.44	− 0.035	0.00
C_3_H_4_O	− 0.453	3.01	0.017	0.00	-0.399	2.73	0.032	0.00	− 0.412	3.11	− 0.102	0.00	− 1.92	1.19	− 0.372	1.24	− 1.002	1.16	− 0.142	0.00
C_3_H_8_O	− 0.434	1.85	− 0.07	0.00	− 0.355	1.78	− 0.08	0.00	− 0.450	2.44	− 0.291	0.00	− 0.586	1.67	− 0.127	1.06	− 0.826	1.02	0.059	0.00
C_4_H_8_O	− 0.534	1.91	− 0.015	0.00	− 0.416	2.18	− 0.04	0.00	− 0.480	2.31	− 0.223	0.00	− 1.033	1.40	− 0.389	1.23	− 0.772	1.7	− 0.094	0.00

**Table 2:** Our calculated Adsorption energies (eV) of air interfering molecules (N_2_, O_2_, CO_2_, H_2_O) on three MXenes of interest are shown and compared to data available in literature for sake of benchmarking.

	Ti_3_C_2_O_2_	Ti_3_C_2_F_2_	Ti_3_C_2_S_2_	Literature
N_2_	− 0.145	− 0.131	− 0.119	− 0.170^A^, − 0.130^B^, − 0.120^C^, − 0.160^D^
O_2_	− 0.966	− 0.125	− 0.124	− 0.126^E^, − 0.08^C^, − 0.01^D^
CO_2_	− 0.209	− 0.197	− 0.196	− 0.20^B^, − 0.21^F^, − 0.14^C^, − 0.21^D^
H_2_O	− 0.199	− 0.166	− 0.164	− 0.21^B^

^A^ Ref.^30^, ^B^ Ref.^31^, ^C^ Ref.^32^, ^D^ Ref.^33^, ^E^ Ref.^34^, ^F^ Ref.^35^.

**Figure 3:**
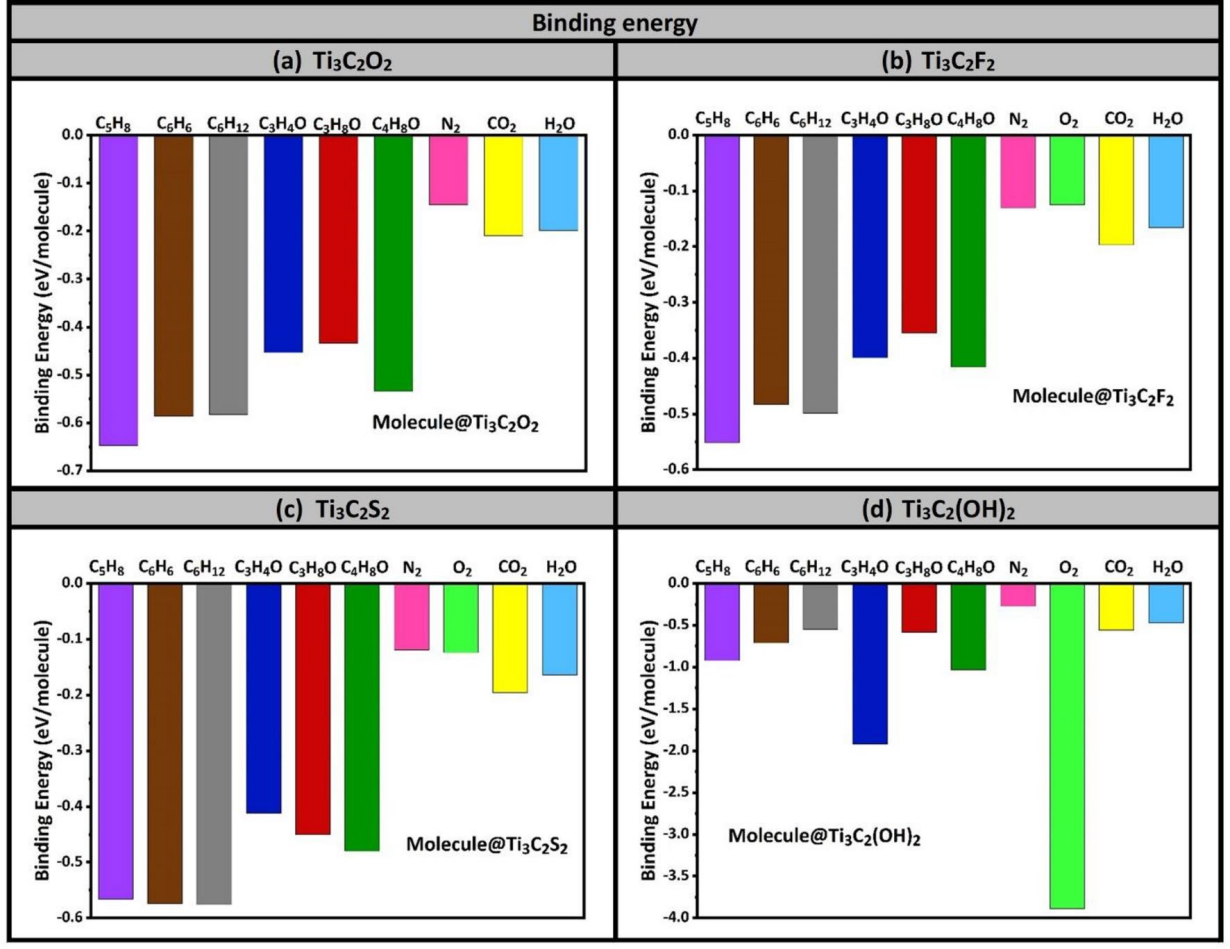
Binding energies of 10 molecules deposited on four Ti_3_C_2_T_x_ MXenes functionalized with T = O, F, S, and OH group. 10 molecules comprise six VOCs lung-cancer biomarkers and four air molecules existing in healthy exhaled breath (i.e., N_2_, O_2_, CO_2_, H_2_O). If E_ads_ > 0, then it should indicate that the molecule does not bind to the surface of MXenes.

The results of adsorption energies of the 10 molecules (six VOCs and four air molecules) on four MXenes (i–iv) are displayed in Fig. 3. Furthermore, as being potential candidates for the selective detection of biomarkers, only three MXenes are selected for the rest of investigation: (i) Ti_3_C_2_O_2_, (ii) Ti_3_C_2_F_2_, and (iii) Ti_3_C_2_S_2_. One more remark about the behavior of oxygen molecule with Ti_3_C_2_O_2_ MXene, as it is shown in the bar chart diagram, O_2_ has a positive adsorption energy indicating that Ti_3_C_2_O_2_ is more invulnerable to oxidation. This trend is also consistent with the finding of many other ab-initio simulations existing in literature^32–34^ (see Table 2). Although our current findings reveal that the O_2_ molecule does not get adsorbed on Ti_3_C_2_O_2_ MXene, yet this trend is still in favor of aiming the selectivity towards the VOCs biomarkers. So, the oxygen data is just excluded in Fig. 3a.

For the case of Ti_3_C_2_O_2_, the logical rational contribution of vdW physical interactions has been evaluated. Intriguingly, our findings revealed that intrinsic vdW interactions account for 100% of the binding energy and are the main contributors to it. For instance, the VOCs were found to exhibit a positive adsorption energy (i.e., not interacting with the substrate) upon the switching off the vdW interactions in our simulations.

To demonstrate the selectivity of the detection of VOCs, the adsorption energies of four interfering air molecules (N_2_, O_2_, CO_2_, and H_2_O) are included in Table 2 and compared to those existing in literature. Our results are in excellent agreement with literature. For instance using Ti_3_C_2_O_2_ MXenes, our results of adsorption energies ${E}_{ads}$ = -0.145 eV, + 0.966 eV, -0.209 eV and -0.199 eV for the respective air molecules compare favorably with the values reported in the literature of ranges [-0.17,-0.12] eV^30–33^, [-0.12,-0.01] eV^32–34^ of weak binding, [-0.23,-0.14] eV^31–35^, and -0.21 eV^31^, respectively.

### Spin polarized density of states (DOS)

**Figure 4:**
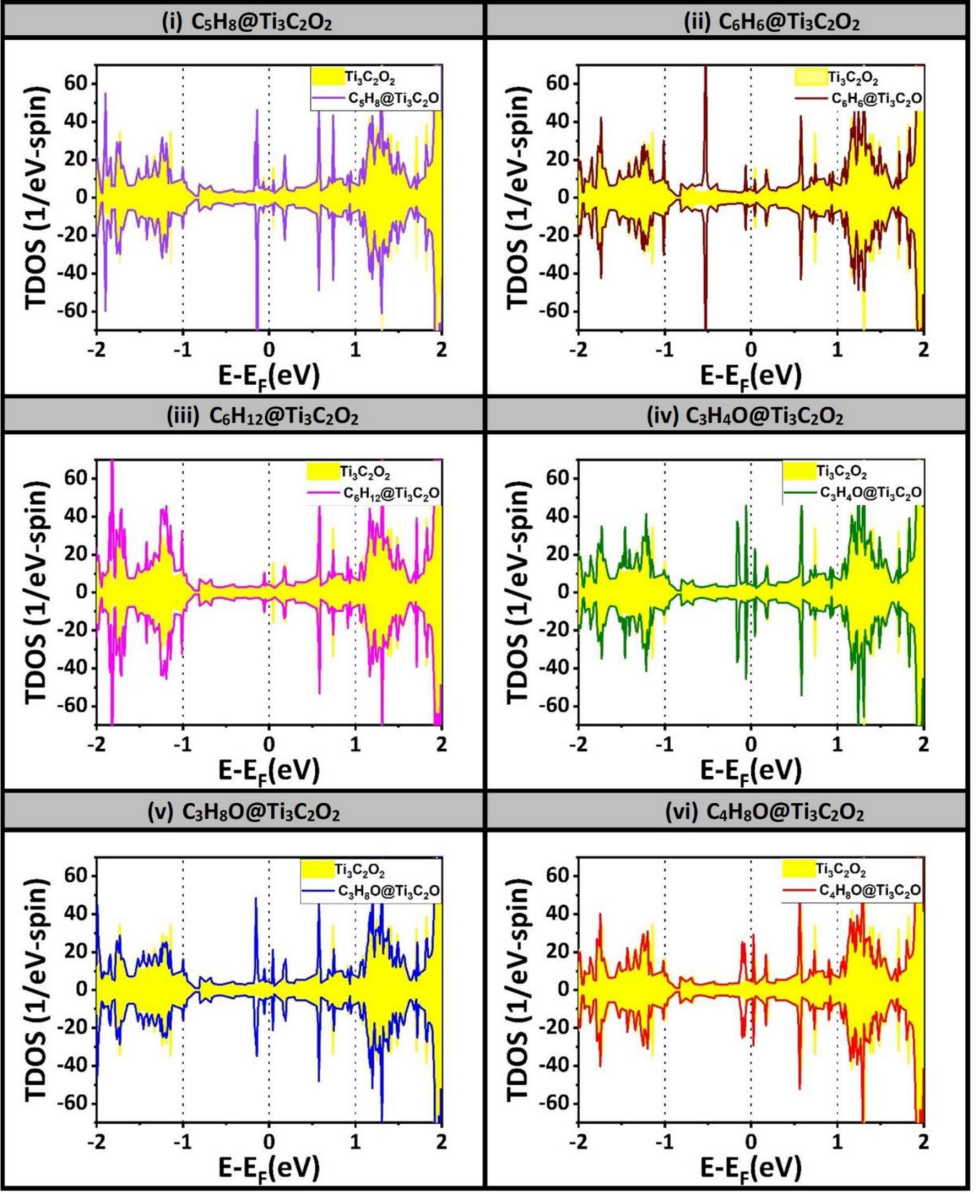
Spin-polarized TDOS of 6 systems due to the interactions of 6 VOCs lung-cancer biomarkers with Ti_3_C_2_O_2_ MXenes. Shaded curves are due to the substrate whereas the solid curves are attributed to the VOC-MXenes systems. Fermi level is take as an energy reference (E_F_ = 0).

Figure 4 shows the spin-polarized total density of states (TDOS) of Ti_3_C_2_O_2_ MXenes after the adsorption of the six VOCs in solid lines. The spin-polarized TDOS of MXenes without the VOCs is shown in shaded curve. The Fermi level is taken as an energy reference (i.e., E_F_ = 0). All the results of TDOSs show that the MXenes exhibit metallic characters which is attributed to the d state of the titanium atoms (Ti). Subsequently, the difference between solid and shaded curves especially at the energy region near Fermi level should reveal the effect on transport rectification and consequently the sensor response. Focusing on the discrepancies in the region near Fermi level of Ti_3_C_2_O_2_, one may notice their existences in VOCs biomarkers number i–iii (i.e., Isoprene “C_5_H_8_”, Benzene “C_6_H_6_”, and Cyclohexane “C_6_H_12_”). Figure S1 (supplementary documents) displays the spin-polarized TDOS of other two MXenes, which are also selected to be candidates for selective gas-sensing of VOCs biomarkers. Namely, Figure S1-a and S1-b corresponding to MXenes Ti_3_C_2_F_2_ and Ti_3_C_2_S_2_ before and after the adsorption of six VOCs biomarkers. Once again, by focusing at energy region around Fermi level, one can notice some discrepancies between shaded and solid curves, especially being very pronounced in the three VOCs biomarkers on Ti_3_C_2_S_2_ MXenes. Yet, these results of TDOS need further analysis to make them more useful. The calculations of the conductivity, using the Drude model, should follow below, as well as charge transfer through both Bader charge analysis and charge density difference (CDD).

### Charge transfer and sensor response

**Figure 5:**
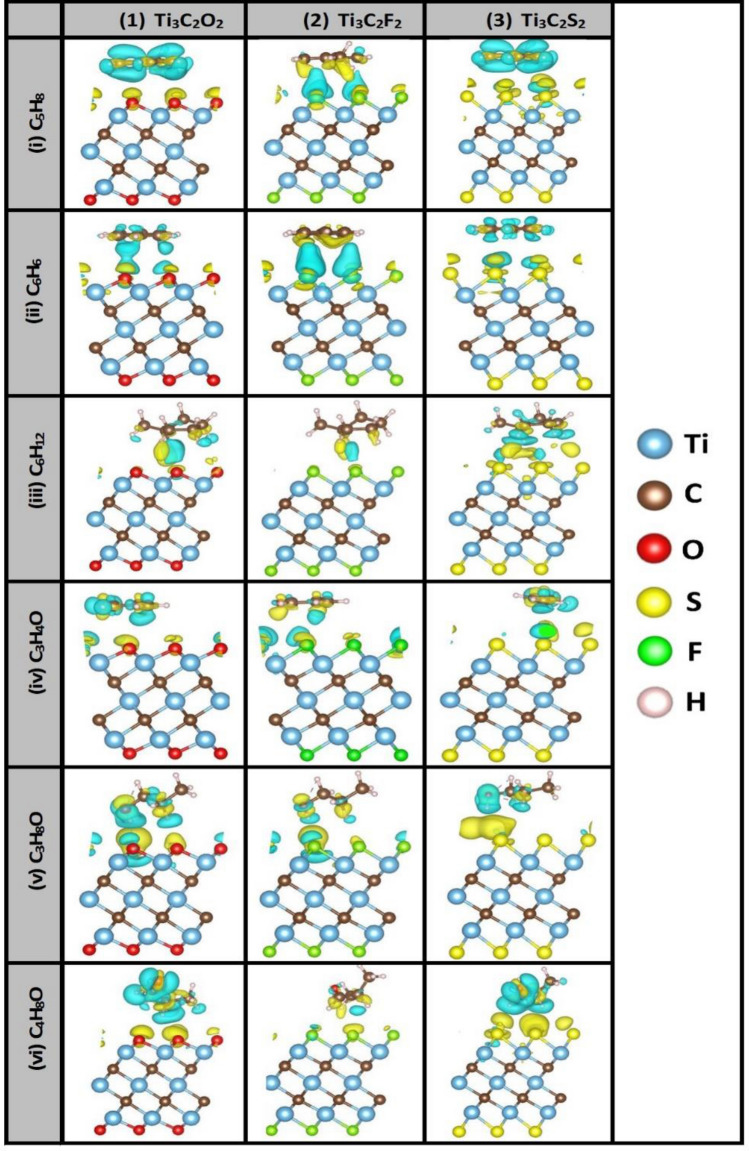
Charge density difference (CDD) of 6 VOCs lung-cancer biomarkers after getting relaxed on 3 Ti_3_C_2_T_x_ MXenes functionalized with T = O, F, or S. Colors of atoms: Ti (sky blue), C (brown), O (red), and H (white). Gain (deficit) of charge is indicated by color yellow (cyan).

Figure 5 shows the results of charge-density difference (CDD) of six VOCs biomarkers on three MXenes: (i) Ti_3_C_2_O_2_, (ii) Ti_3_C_2_F_2_, and (iii) Ti_3_C_2_S_2_. Charge gain (deficit) is shown in yellow (cyan) color. Only side views are shown for sake of clarity. In most of the cases, the VOCs biomarkers are acting as oxidizing molecules by attracting the charges from the passivation layers. Namely, **(1)** On Ti_3_C_2_O_2_ MXene, three of the VOCs are found to attract charges from oxygen layer, in other words “oxidizing” (i.e. Cyclohexane “C_6_H_12_”, Propanol “C_3_H_8_O”, and Butyraldehyde butanal “C_4_H_8_O”) while the other three found “reducing” (i.e., Isoprene “C_5_H_8_” and Benzene “C_6_H_6_, and Cyclopropenone “C_3_H_4_O”), where the overall interactions with the substrate resulted in donating the oxygen passivation layer some charges. **(2)** On Ti_3_C_2_F_2_ MXenes, the VOCs look like oxidizing by attracting just small amounts of charge from the fluorine layer. **(3)** On Ti_3_C_2_S_2_ MXenes, the charge transfer is well pronounced for almost all the VOCs as acting to be reducing and giving out some charges to the sulfur layer.

**Figure 6:**
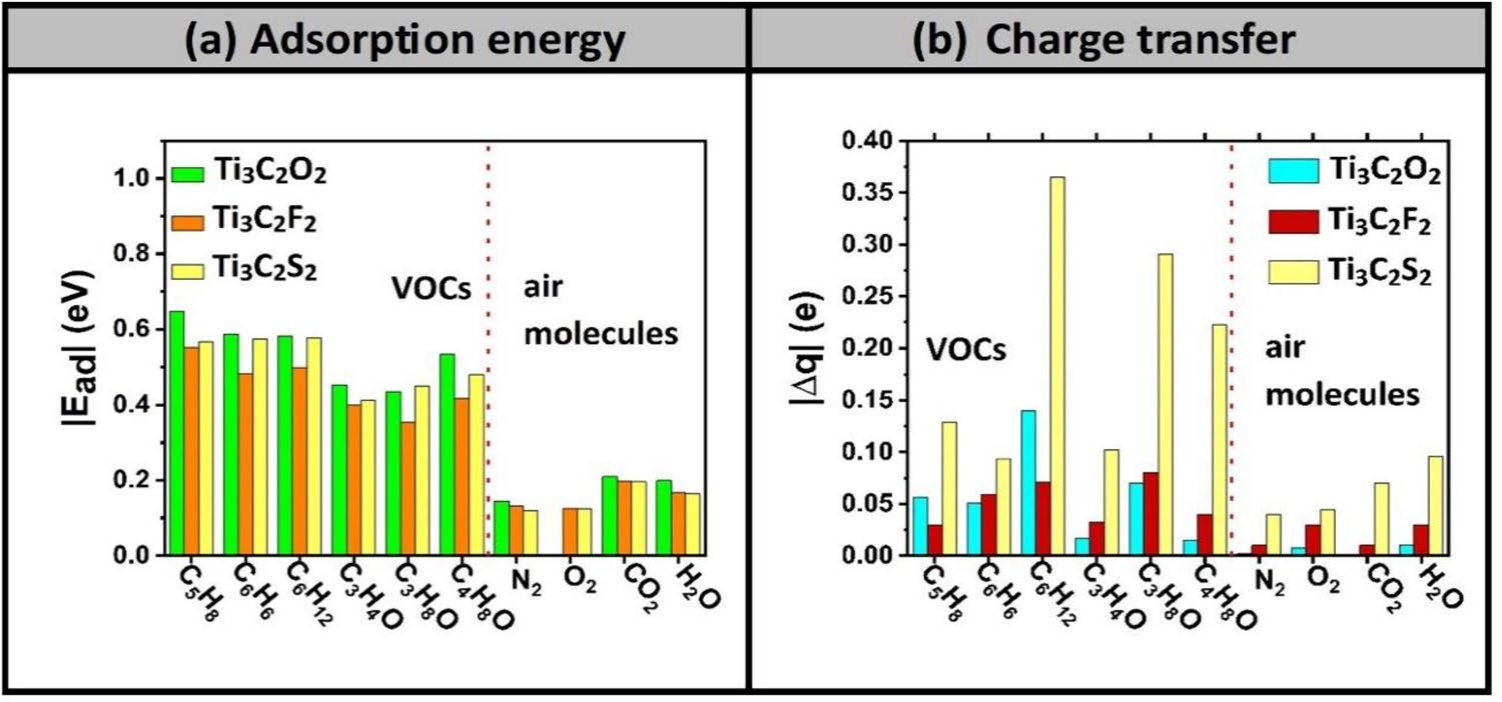
adsorption energy (**a**) and charge transfer (**b**) attributed to the relaxation of 10 molecules (comprising 6 VOCs lung-cancer biomarkers + 4 air molecules interfering the healthy exhaled breath) on 3 Ti_3_C_2_T_x_ MXenes functionalized with T = O, F, or S.

Figure 6 shows the results of both (a) the absolute values of the adsorption energies and (b) charge transfers, which are originally summarized in Table 1 for the adsorption processes of 10 molecules (i.e., six VOCs biomarkers + four air molecules) on the three MXenes (1) Ti_3_C_2_O_2_, (2) Ti_3_C_2_F_2_, and (3) Ti_3_C_2_S_2_. From the perspective of the adsorption energies, VOCs biomarkers possess much higher values than the four interfering air molecules existing in the exhaled breath (N_2_, O_2_, CO_2_ and H_2_O). Whereas, from the perspective of charge transfer, the values are larger on the side of VOCs biomarkers but not as pronounced as the adsorption energies.

**Figure 7:**
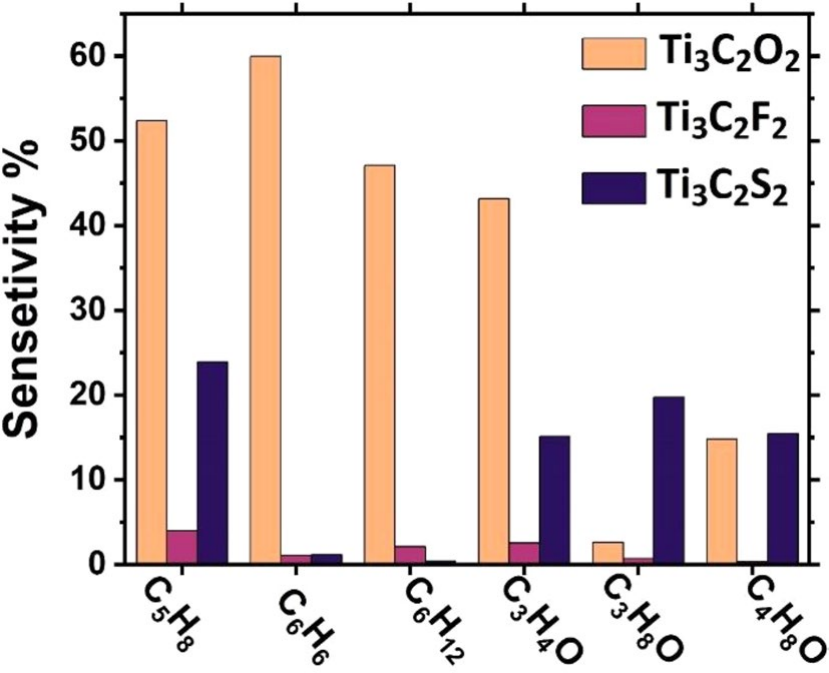
Sensor responses of 6 VOCs lung-cancer biomarkers after their adsorption on 3 Ti_3_C_2_T_x_ MXenes functionalized with T = O, F, or S. Ti_3_C_2_O_2_ MXenes is shown to have distinguished selectivity toward at least four VOCs biomarkers. Thus, it should be the best candidate for platform of reusable biosensor with promising early diagnosis of lung cancer diseases.

Figure 7 shows the sensor response for the six VOCs on the three MXenes (1) Ti_3_C_2_O_2_, (2) Ti_3_C_2_F_2_, and (3) Ti_3_C_2_S_2_. The results confirmed that Ti_3_C_2_O_2_ MXenes to be the best candidate for the selective sensing of at least four VOCs biomarkers (namely: isoprene “C_5_H_8_”, benzene “C_6_H_6_”, cyclohexane “C_6_H_12_”, and cyclopropanone “C_3_H_4_O”), which mostly act as reducing agents. The sensor response for these four molecules on Ti_3_C_2_O_2_ MXenes are ranging from 52%, 60%, 47%, to 43%, respectively, as shown in Table S1. One recalls that the direct-current (DC) conductivity was estimated using the Drude formula, which is valid for a system of quasi-free electron gas, as in case of MXenes which have metallic characters. So, it is concluded that Ti_3_C_2_O_2_ to be the best candidate for detecting four VOCs biomarkers.

In order to assess whether the sensor is useable or disposable, we have calculated the recovery time using Eq. (4) at room temperature and under the exposure of visible light. The results are shown in Table 3 in units of seconds. It is remarkable that the recovery time for our selected MXenes (i.e., O-, F-, and S-passivated Ti_3_C_2_ MXenes) is small and ranging between μs and ms. Such results are very encouraging and rather corroborating the suitability of these MXenes for the selective detection of the studied VOCs as they provide moderately strong physisorption interactions to alter the transport properties and sensor response while maintaining the recovery time small enough. So, they can be good candidates for platforms in reusable biosensors for efficient detection of lung cancer biomarkers.

**Table 3:** Recovery time of VOCs’ desorption on the selected MXenes (unit of second), calculated at room temperature and exposed to visible light.

	Ti_3_C_2_O_2_	Ti_3_C_2_F_2_	Ti_3_C_2_S_2_	Ti_3_C_2_(OH)_2_	Ti_3_C_2_F(OH)
C_5_H_8_	7.41 × 10^−3^	1.81 × 10^−3^	3.23 × 10^−3^	3.21 × 10^+3^s = 0.89 Hr	2.81
C_6_H_6_	7.00 × 10^−3^	1.30 × 10^−4^	4.40 × 10^−3^	0.95	0.028
C_6_H_12_	6.00 × 10^−3^	2.42 × 10^−4^	4.75 × 10^−3^	1.67 × 10^−3^	4.94 × 10^−3^
C_3_H_4_O	4.08 × 10^−5^	5.05 × 10^−6^	8.35 × 10^−6^	1.80 × 10^+20^	6.82 × 10^+4^
C_3_H_8_O	1.96 × 10^−5^	9.20 × 10^−7^	3.63 × 10^−5^	7.00 × 10^−3^	75.38
C_4_H_8_O	9.36 × 10^−4^	9.70 × 10^−6^	1.16 × 10^−4^	2.26 × 10^+5^	9.33

## Conclusions

An ab-initio method based on VASP was employed to search for the suitable functionalization of Ti_3_C_2_-based MXenes as platform for biosensor for selective detection of lung cancer biomarkers. Six VOC biomarkers {namely, (i) isoprene “C_5_H_8_”, (ii) benzene “C_6_H_6_”, (iii) cyclohexane “C_6_H_12_”, (iv) cyclopropanone “C_3_H_4_O”, (v) propanol “C_3_H_8_O”, and (vi) butanal “C_4_H_8_O”} versus four interfering air molecules {i.e., N_2_, O_2_, CO_2_, and H_2_O} were considered in the investigation of the adsorption, transport and gas sensing properties. While all molecules were found to exhibit physisorption processes on the studied Ti_3_C_2_T_x_ MXenes (with T_x_ = O, F, S, (OH), or F(OH)) groups), the interactions between the VOC biomarkers and, particularly, the O-, F-, and S-passivated MXenes are found to be relatively strong (with ${E}_{ads}\cong -0.45 to-0.65 eV$) and to yield higher sensitivity. The reason behind such enhanced physisorption is attributed to the formation of strong electric dipole moments in the VOCs which in turn strengthen the van der Waals interactions to the extend to affect the states near Fermi level of the adsorbent. Such effects can rectify the conductivity and consequently the sensor response. Furthermore, such scenario or trend is more pronounced in the case of interaction between the O-passivated Ti_3_C_2_ MXenes and the O-containing VOCs. Equally importantly, the calculated recovery time at room temperature under the visible light exposure was found small at the order ranging between few μs to ms. It is concluded that, among the studied systems, Ti_3_C_2_O_2_ was the best candidate as platform of efficient reusable biosensor the selective detection of lung cancer biomarkers, thus, enabling early diagnosis of lung cancer diseases.

### Computational methodology

The computational supercell is composed of 3 × 3 primitive cells (PCs) of Ti_3_C_2_T_x_ MXenes with periodic boundary conditions applied along the x and y directions and vacuum space of 20 Å along the z direction to enable the simulation of a single isolated monolayer (ML). The passivation layer on Ti_3_C_2_T_x_ MXenes, in both side and top views, are shown in Fig. 1. Pristine Ti_3_C_2_ MXenes has a triangular lattice constant a = 3.050 Å, which is in good agreement with the experimental value of 3.057 Å^36^. Lattice parameters of Ti_3_C_2_T_x_ MXenes with T_x_ = O, F, S, and OH are a = 3.027, 3.062, 3.124 Å, and 3.067 Å, respectively. Accordingly, for instance the supercell of Ti_3_C_2_O_2_ has the dimensions A = B = 9.081 Å, C = 30 Å and contains 63 atoms (27 Ti + 18 C + 18 O atoms). Furthermore, the VOCs biomarkers were also relaxed in their free standing states and the results of relaxations are displayed in Fig. 1. The relaxed structures are found to be in good agreement with literature vis-à-vis morphology and bond lengths^13^.

The computational method is based on DFT as implemented in VASP package. This code is worldwide popular and very reliable in predicting the ground state properties of solids and molecules and should be the most suitable for the adsorption properties. It solves the Kohn–Sham equations iteratively using plane wave basis set with periodic conditions. Projected-augmented plane waves were employed to describe the electron–ion interaction^37^. The exchange and correlation interaction is handled using the Perdew-Burke-Ernzerhof formula of the generalized gradient approximation (GGA) approach^38^. Plane wave basis set is used up to an energy cut-off of 520 eV. The sampling of the Brillouin zone is carried out using the special-k point technique due to the Monkhorst–Pack scheme^39^. For the atomic relaxations and density of states calculations, grids of 4 × 4 × 1 and 6 × 6 × 1 were used, respectively. In the DFT self-consistent cycle, convergence criteria of 10^–6^ eV on total energy and 0.01 eV/Å on force per atom were used. The inclusion of van der Waals (vdW) interaction is essential and was implemented using DFT-D3 method of Grimme scheme^40^. The atomic charge transfers are evaluated using the Bader charge analysis^41^.

To study the adsorption, the VOCs and interfering air molecules were individually brought close to the surface of functionalized MXenes within a distance of about 2.0 Å and atomic relaxations started. Initial configurations of molecules on different sites and in different orientations were tested. The results of total energy calculations are explored to calculate the molecular adsorption energy:

1 $${E}_{ads}={E}_{tot}^{MXene-Mol}-{E}_{tot}^{MXene}-{E}_{tot}^{Mol}$$

where ${E}_{tot}^{MXene-Mol}$, ${E}_{tot}^{MXene}$, and ${E}_{tot}^{Mol}$ are total energies of molecule adsorbed on MXene, MXene, and the free molecule, respectively. The ratio (R) of the contribution of vdW interactions to the molecular adsorption energy can be evaluated using the following formula:

2 $$R=\frac{\left|{E}_{ads}^{vdW}-{E}_{ads}^{NovdW}\right|}{\left|{E}_{ads}^{vdW}\right|}\times 100\%$$

where ${E}_{ads}^{vdW}$ and ${E}_{ads}^{NovdW}$ are the adsorption energies with and without vdW interactions, respectively.

After the adsorption process, the MXenes maintain their metallic characters. So, one can evaluate the DC conductivity using Drude formula (see reference^42^ for details). Then, the sensor response can be estimated as follows^42^:

3 $$S=\frac{\left|\overline{{N }_{F}^{aft}}-\overline{{N }_{F}^{bef}}\right|}{\overline{{N }_{F}^{bef}}}\times 100$$

where $\overline{{N }_{F}^{bef}}$ and $\overline{{N }_{F}^{aft}}$ are the density of states at Fermi level before and after the molecular adsorption, but averaged in an energy range [*E*_*F*_-0.3,*E*_*F*_ + 0.3] eV around Fermi level.

In order to decide about whether the sensor should be reusable or disposable, one may estimate the recovery time^43^ given by:

4 $$\tau ={\upsilon }_{o}^{-1}{\text{exp}}\left[-\frac{{E}_{ads}}{{k}_{B}T}\right]$$

where ${\upsilon }_{o}$ is the attempt frequency factor, having values 10^12^ and 10^16^ Hz under visible and UV light situations, respectively^44,45^. E_ads_ is the adsorption energy, k_B_ and T are the Boltzmann constant and the absolute temperature, respectively.

### Supplementary Information

Supplementary Information.

## Data Availability

The datasets generated and/or analyzed during the current study are available from the corresponding author on reasonable request.
