# Supplementary material for: Ab initio investigation of functionalization of titanium carbide Ti3C2 MXenes to tune the selective detection of lung cancer biomarkers
Source: Sci Rep. 2024 Jan 16;14:1403. doi: 10.1038/s41598-024-51692-6 (PMC10791681; doi:10.1038/s41598-024-51692-6)
Supplement: Supplementary file 1 — Supplementary Information. [file 41598_2024_51692_MOESM1_ESM.docx]

**Supplementary Documents**

**Table S1:** Sensor responses of six VOCs lung-cancer biomarkers after adsorption processes on five Ti_3_C_2_T_x_ MXenes functionalized with T = O, F, S, (OH), or F(OH) groups, respectively.


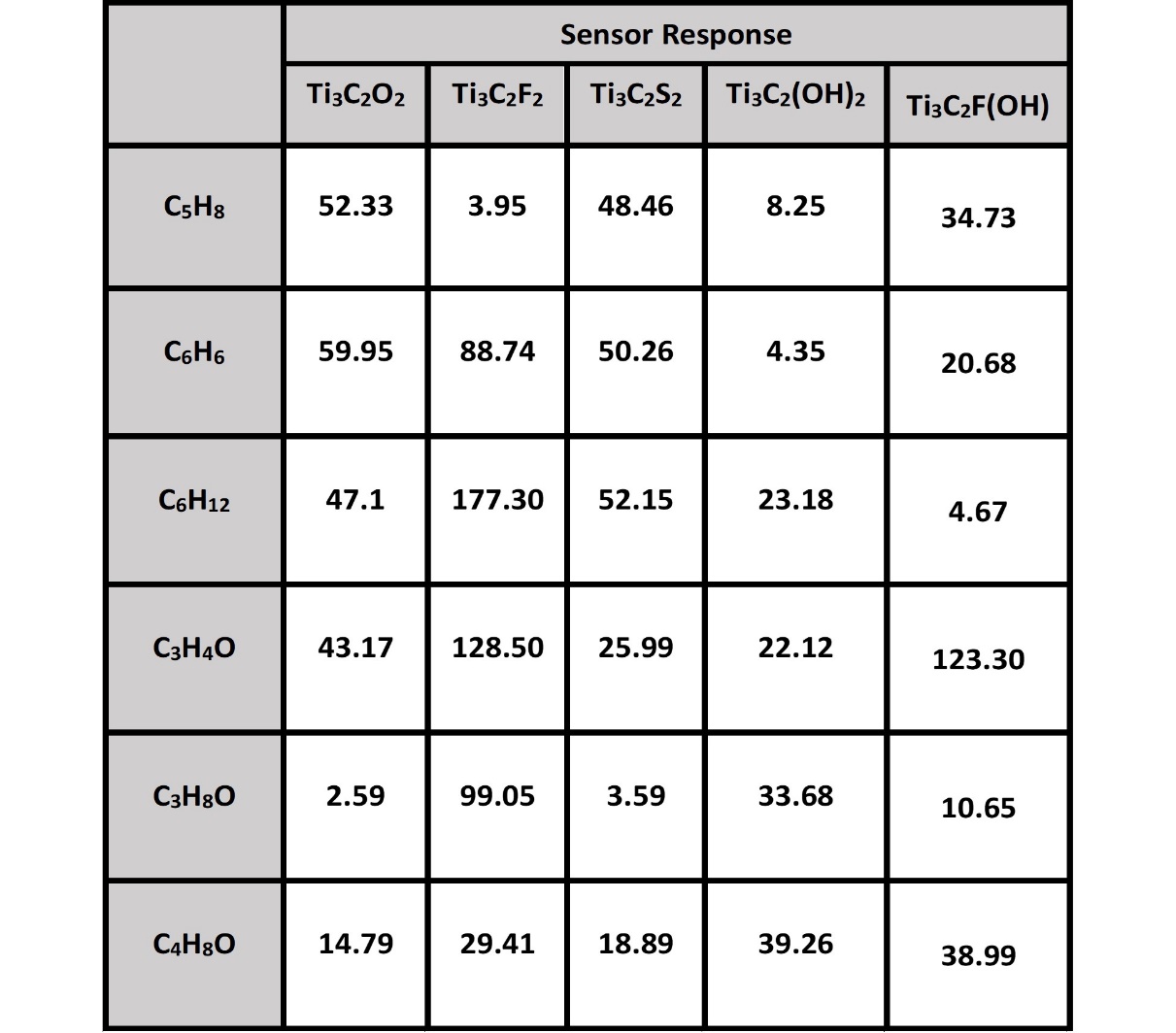


**
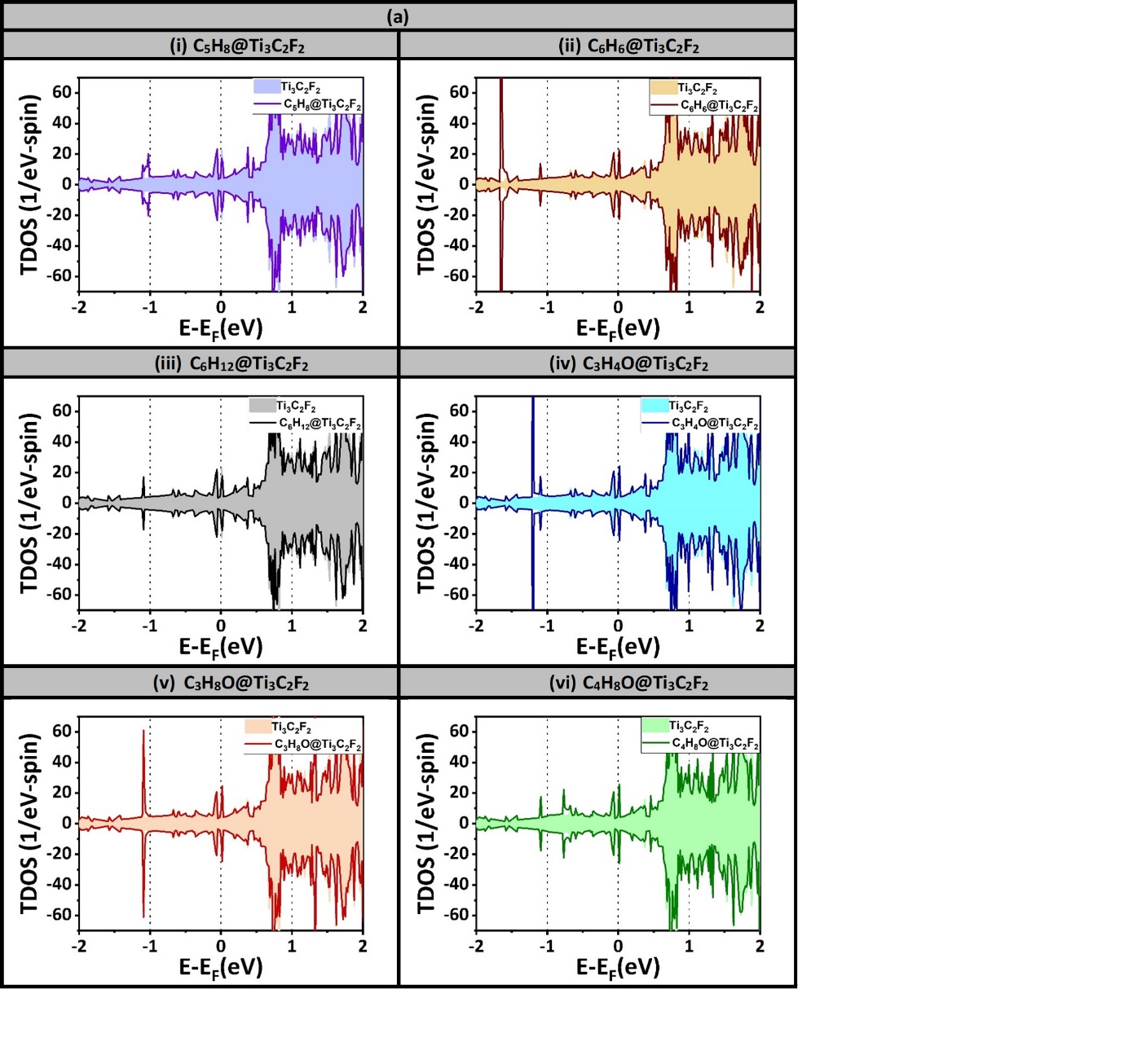
**

**
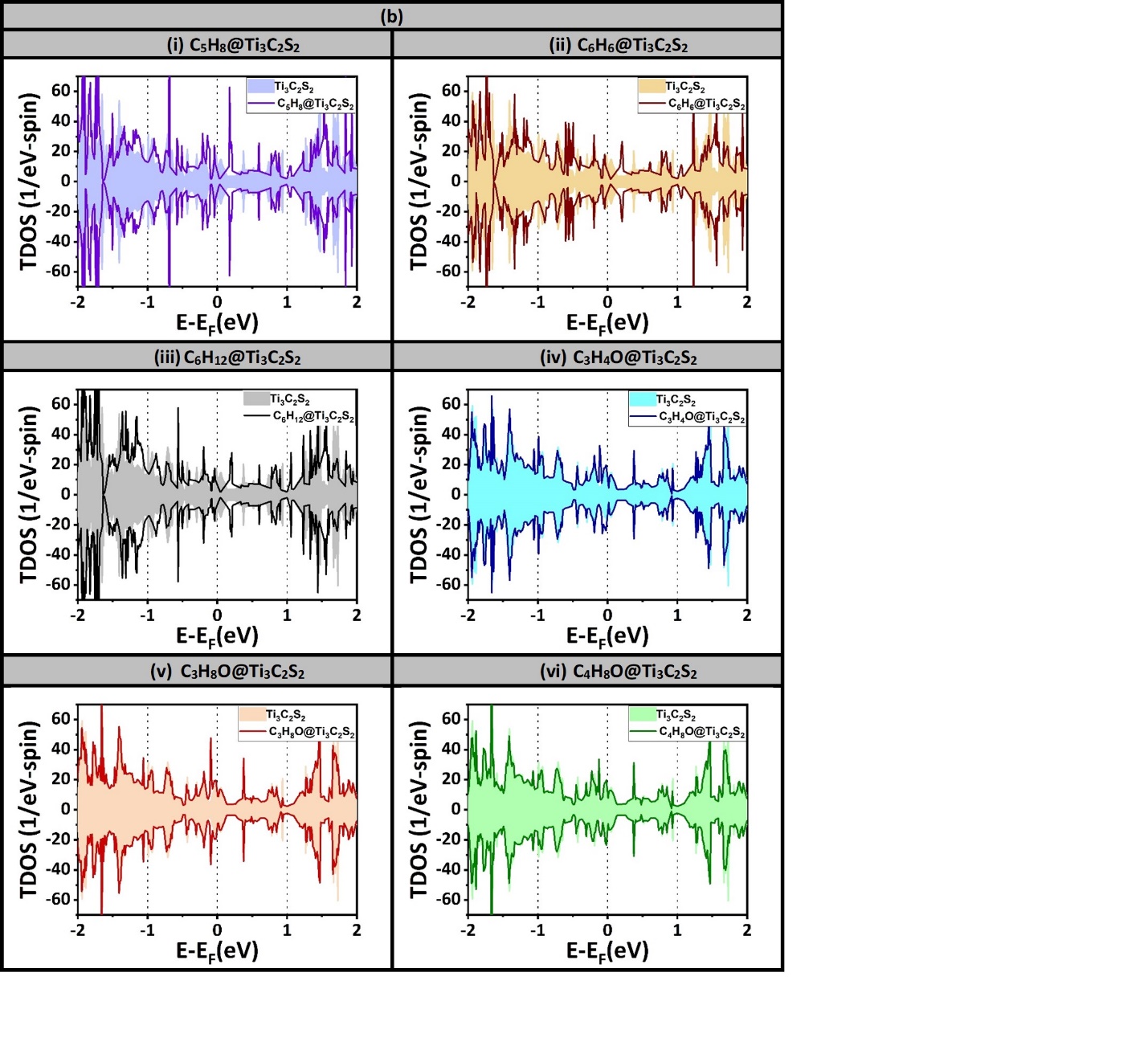
**

**Figure S1:** Spin-polarized TDOS of 6 systems due to the interactions of six VOCs lung-cancer biomarkers with two Ti_3_C_2_T_x_ MXenes: (a) Ti_3_C_2_F_2_ MXenes and (b) Ti_3_C_2_S_2_ MXenes. Shaded curves are due to the substrate whereas the solid curves are attributed to the VOCs-MXenes systems. Fermi level is take as an energy reference (E_F_ = 0).
